# Disparities in the incidence and prevalence of psychotic disorders among people with and without disabilities in south Korea: A national database study

**DOI:** 10.1186/s13690-025-01691-4

**Published:** 2025-08-04

**Authors:** Kyoung Eun Yeob, So Young Kim, Yeon Yong Kim, Jong Hyock Park

**Affiliations:** 1 Institute of Health and Science Convergence, Chungbuk National University, Cheongju, Republic of Korea; 2 Department of Public Health and Preventive Medicine, Chungbuk National University Hospital, Cheongju, Republic of Korea; 3 Big Data Steering Department, National Health Insurance Service, Wonju, Republic of Korea; 4 Drug Evaluation Department, National Institute of Food and Drug Safety Evaluation, Cheongju, Republic of Korea; 5 Department of Preventive Medicine, College of Medicine, Chungbuk National University, Cheongju, Republic of Korea; 6 Department of Public Health and Preventive Medicine, Chungbuk National University Hospital, Cheongju, Republic of Korea

**Keywords:** Schizophrenia, Disability, Disparity, South korea, Big data

## Abstract

**Background:**

Numerous epidemiological studies on psychotic disorders have been conducted, however, most were from an etiological perspective, used small sample sizes, or focused on a limited number of disabilities. Few studies have investigated all types of disabilities over a long observation period, and none have included the entire adult population of a country. To explore potential differences in the incidence and prevalence of psychotic disorders between patients with and without disabilities, we conducted a serial cross-sectional study.

**Methods:**

This study was conducted using a data set linking the Korean National Health Insurance Service database, and disability registration data. Age-standardized incidence or prevalence rates were calculated for each year during 2008–2017 according to the presence, severity, and type of the disability. Factors associated with psychotic disorders were examined by multivariate logistic regression using the most recent data.

**Results:**

The age-standardized incidence and prevalence of psychotic disorders were higher among people with disabilities than among those without disabilities across all age groups, with particularly high rates observed among people with severe disabilities and those with intellectual disabilities. The gap in the prevalence of psychotic disorders between people with and without disabilities has widened over time. In regression analyses, adjusting for sociodemographic characteristics and mental health substantially reduced the odds ratios.

**Conclusions:**

The incidence and prevalence of psychotic disorders were higher in people with disabilities, especially those with severe disabilities, and intellectual disabilities. Policy efforts are needed to narrow the gap people with and without disabilities.

**Supplementary Information:**

The online version contains supplementary material available at 10.1186/s13690-025-01691-4.

**Table:** 

**Text box 1. Contributions to the literature**	
• There is limited evidence on the incidence and prevalence of psychotic disorders in people with disabilities, particularly from studies encompassing national populations.	
• The incidence and prevalence of psychotic disorders were higher in people with disabilities, with the difference being especially pronounced among those with intellectual disabilities.	
• The gap in prevalence between people with and without disabilities was larger than the gap in incidence.	
• Important reasons for these finding may be the ‘low socioeconomic status’ and ‘mental health’ of people with disabilities.	

## Introduction

The 21st century has brought about a burgeoning recognition of the central role of mental health and illness to overall population health [1]. The emphasis of these public mental health efforts has largely been on mental wellbeing and common mental disorders, such as depression, anxiety, and substance use disorders. Even though psychotic disorders, which include schizophrenia, bipolar disorder, and substance-induced psychoses, are increasingly recognized as an important public health issue [1]public health has less focus on rare conditions.

Psychotic disorders are mental health illnesses characterized by an impaired relationship with reality, usually with associated behavioral changes [2]. Psychotic disorders can be highly distressing, impacting a person’s quality of life and ability to maintain self-care, making it difficult fora person to keep up with employment, and maintain relationships and a social life [3]. The social stigma caused by psychotic disorders can affect life and the lives of family members, moreover, people who have a psychotic condition may experience suicidal ideation (thinking about suicide) or might attempt suicide [4]. Despite being a rare condition [5]schizophrenia is globally one of the top 20 leading contributors to years lived with disability [6]high disability weighting, and chronic course of illness [7]. Previous research for the causes of schizophrenia has predominantly originated from two research paradigms; ‘Genetics’ and ‘Epidemiology’ [8]. Although each approach has made important contributions to etiological understanding, neither has fully resolved the exact milieu of risk factors for schizophrenia from a public health perspective.

Given that people with disabilities are more likely to experience concurrent psychological problems [9–11], it may be anticipated that psychotic disorders may have a greater impact on individuals with disabilities compared to those without disabilities. Previous studies have reported a higher prevalence of schizoid behavior among intellectual disabilities (ID) [12, 13] or individuals who have experienced severe traumatic brain injuries [14]. Numerous epidemiological studies on psychotic disorders have been conducted, however, most of the studies were conducted from an etiological perspective, and conducted with small sample sizes, or only on a limited number of disabilities; relatively few focused on all types of people with disabilities, especially with a long observation period [6, 12, 13, 15]. Moreover, no study has included the whole adult population of a country.

In this study, we analyzed the risk factors for schizophrenia among people with disabilities through the lens of social-epidemiology, and to discuss whether it warrants the design and implementation of strategies of prevention for schizophrenia. Our target population included people with disabilities diagnosed with psychotic disorders; the comparison group included patients with psychotic disorders without disabilities and the outcome variable was the incidence and prevalence of psychotic disorders. To explore potential differences in the incidence and prevalence of psychotic disorders between patients with and without disabilities, we conducted a serial cross-sectional study.

## Methods

### Data source and study subjects

We linked two population datasets for our study. The dataset from the Korean National Health Insurance Service (NHIS) was linked with the Disability Registration System to accurately identify individuals with disabilities. The NHIS data provided comprehensive healthcare utilization and diagnosis information, while the Disability Registration System enabled classification by disability type and severity.

***Korean National Health Insurance Service*** Information was obtained from the National Health Insurance Service (NHIS) database of the National Health Insurance Sharing Service. The National Health Insurance (NHI) is a single-payer social health insurance system that is mandatory for all residents of Korea; thus, the NHI claims DB covers the entire population of South Korea (over 50 million) [16]. The NHIS has information pertaining to age, gender, residential area, monthly insurance contributions (a proxy for income status), and vital statistics. The NHIS claims database enables easy retrieval and analysis of population-based epidemiological data.

***Disability Registration System in the Republic of Korea*** We collected information on disability severity and type from a disability registry. The database covered 93.8% of the total disabled population as of 2011 [17]. Using Korean personal identification numbers, disability severity and type were linked with variables selected from the NHIS claims database.

For this study, population-based medical data for adults (older than 19 years) with psychotic disorders were retrospectively extracted from the NHI claims database from January 2008 to December 2017. We excluded people with psychiatric disabilities from the analysis. People with psychiatric disabilities refer to persistent and severe psychiatric conditions such as schizophrenia that result in long-term functional impairments. Because these conditions overlap substantially with our outcome of interest-namely, the incidence and prevalence of psychotic disorders-we excluded people with psychiatric disabilities from the analysis to avoid conceptual redundancy and ensure a more appropriate comparison between people with and without disabilities.

### Definition of psychotic disorder

The following outcome variables were used: the incidence and prevalence of psychotic disorders. Diagnosis codes in the claims database are based on the International Classification of Diseases, 10th revision (ICD-10), which supports the validity of disease classification. These diagnoses are subject to review under the National Health Insurance claims review system, which applies standardized criteria across all healthcare institutions, thereby enhancing the reliability of the data. Furthermore, as the NHIS database covers the entire Korean population, the use of this nationwide administrative dataset ensures high representativeness and strengthens the external validity of the findings. The incidence of psychotic disorders was defined as the proportion of those who had no record of medical visits for problems with the disease codes F20-F29 (Schizophrenia, schizotypal and delusional disorders) for 2 years prior to the relevant year but were newly diagnosed with the disease in the relevant year. The prevalence of psychotic disorders was defined as the proportion of people who have had more than two outpatient visits or have been hospitalized for treatment of disease codes mentioned above.

### Independent variables

Disability severity was officially scaled from 1 (most severe) to 6 (mildest). Disability types are divided into 15 classifications aligned with the Korean Welfare Act for the Disabled and are detailed in Table S1. We regrouped these into five categories according to previous studies [18, 19]: (1) physical disability (impairment in areas of the body and extremities), (2) brain injury, (3) communication disability (impairments in vision, hearing, and speech), (4) intellectual disability (developmental impairments in cognitive functioning and skills that affect areas such as language, social interactions, and self-care behaviors), and (5) disability due to functional loss in major internal organs (kidney, heart, liver, and respiratory system impairment, and urinary-intestinal fistula, epilepsy, and facial deformity) in the National Disability Registry. The covariates considered in our study included age, gender, income level, type of health insurance, residential area, mental health disease (depression, anxiety and sleep disorder), and the Charlson Comorbidity Index (CCI) for measuring comorbidity.

### Statistical analyses

Descriptive statistics were generated on disability status (present or absent) and the type and severity of disability. Age-standardized incidence and prevalence rates of psychotic disorders were calculated using the 2005 Korean census data as the reference. We examined long-term trends in the incidence and prevalence of psychotic disorders based on the following criteria: (1) disability status (present or absent), (2) disability severity (six grades, and severe vs. mild), (3) disability type (five categories) and (4) combined criteria of severity and type of disability (mild and severe within each of five disability types). The analyses were stratified by gender. To examine the association between disability and psychotic disorders, we analyzed interaction effects in the adjusted multivariate model, and conducted a likelihood-ratio test to compare models with and without interaction terms. Model 1 is a crude odds ratio (OR); model 2 is adjusted for age and gender; model 3 is model 2 + income level, type of health insurance, and residential area; model 4 is model 3 + CCI, and model 5 is model 4 + mental health disease using the most recent dataset available (2017 year). All analyses were performed using SAS software (version 9.3; SAS Institute, Cary, NC, USA), and a p-value < 0.05 was considered significant. This study was approved by the Institutional Review Board of Chungbuk National University (CBNU-202010-HRHR-0717).

## Results

### Study participants

The general characteristics of the two groups are shown in Table 1. In 2017, the number of people with and without disabilities were 2,536,905 (5.9%) and 40,374,667 (94.1%), respectively. Among people with disabilities, 36.7% had severe disabilities and the most frequent type was physical disability (51.8%). People with disabilities generally older than those without disabilities (Over 50 age: 79.3% vs. 43.2%). More people with disabilities lived in rural areas compared to those without disabilities (14.3% vs. 8.6%) and Medicaid was more common (16.7% vs. 2.9%). In total, the incidence of psychotic disorders among individuals without disabilities was 0.1% and 0.2% among those with disabilities. The prevalence of psychotic disorders among individuals with and without disabilities were 9.2% and 1.8%, respectively.

**Table 1:** Demographic characteristics by the presence of disability in 2017

	People without disability		People with disability		*P*-value
	*n*	%	*n*	%	
	40,374,667	94.1	2,536,905	5.9	
Sex					
Male	19,857,936	49.2	1,465,420	57.8	< 0.0001
Female	20,516,731	50.8	1,071,485	42.2	
Age, years					
20–29	6,946,139	17.2	93,845	3.7	< 0.0001
30–39	7,490,860	18.6	143,398	5.7	
40–49	8,501,974	21.1	286,716	11.3	
50+	17,435,694	43.2	2,012,946	79.3	
Income level					
Medical aid and first quartile (lowest)	5,947,722	14.7	410,660	16.2	< 0.0001
Second quartile	6,419,255	15.9	295,924	11.7	
Third quartile	7,389,114	18.3	338,592	13.3	
Fourth quartile	8,494,084	21.0	435,086	17.2	
Fifth quartile (highest)	10,384,899	25.7	598,123	23.6	
Unknown	1,739,593	4.3	458,520	18.1	
Type of insurance					
Medicaid	828,171	2.1	423,915	16.7	< 0.0001
Health insurance	39,546,496	97.9	2,112,990	83.3	
Residence					
Metropolitan	24,787,189	61.4	1,347,851	53.1	< 0.0001
City	11,420,526	28.3	790,104	31.1	
Rural	3,456,954	8.6	362,946	14.3	
Unknown	709,998	1.8	36,004	1.4	
Severity of disability					
Severe			930,825	36.7	
Mild			1,606,080	63.3	
Grade of disability					
1			221,816	8.7	
2			314,668	12.4	
3			394,341	15.5	
4			380,602	15.0	
5			562,732	22.2	
6			662,746	26.1	
Type of disability					
Physical			1,314,177	51.8	
Brain injury			244,136	9.6	
Communication			575,117	22.7	
Intellectual			260,960	10.3	
Major internal organ			142,515	5.6	
Incidence of psycho disorders					
Yes	40,350,823	99.9	2,530,864	99.8	< 0.0001
No	23,844	0.1	6,041	0.2	
Prevalence of psycho disorders					
Yes	39,836,062	98.7	2,302,463	90.8	< 0.0001
No	538,605	1.3	234,442	9.2	

### Long-term trends in the age-standardized incidence of psychotic disorders

**Fig. 1:**
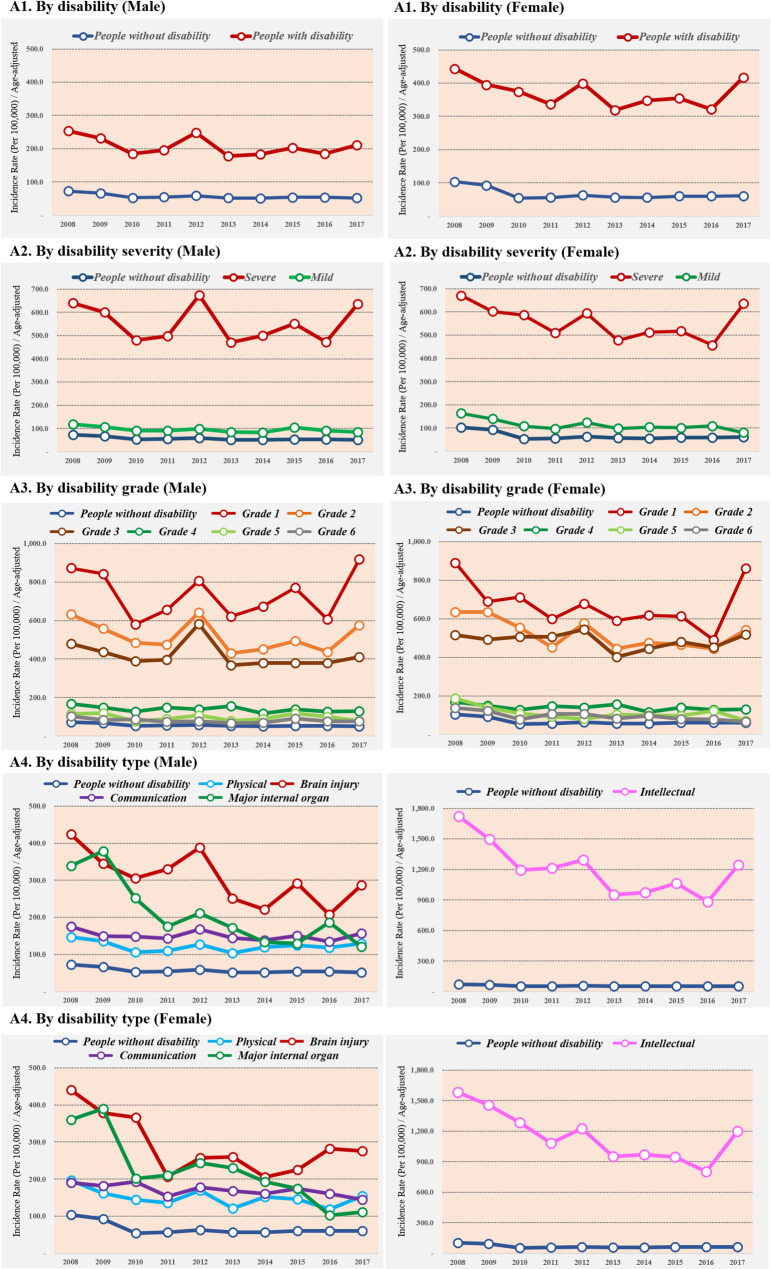
Age-standardized incidence of psychotic disorders by disability characteristics, 2008 to 2017

The trends in age-standardized incidence of psychotic disorders between 2008 and 2017 are shown in Fig. 1. The age-standardized incidence of psychotic disorders was higher among the people with disabilities than among those without disabilities across all ages, the incidence rates were 7.5 times and 6.9 times higher in people with disabilities compared to the people without disabilities in recent years both male and female. Moreover, the incidence in people without disabilities slightly decreased between 2008 and 2017, but increased during the same period in people with disabilities in male group (Average annual percent change (AAPC): -2.8% and 1.0% for non-disabled and disabled).

The trends in age-standardized incidence of psychotic disorders according to the severity of a disability in incidence were complicated. The incidence of psychotic disorders showed a repetitive pattern of increase and decrease, as a result, people without disabilities and people with mild disabilities trends slightly decreased between 2008 and 2017, however, people with severe disabilities remained constant, and grade 1 slightly increased in male groups (AAPC: 0.5% for disabled of grade 1 and − 2.8% for non-disabled).

Among the five types of disabilities, the highest incidence of psychotic disorders was observed among people with intellectual disabilities, followed by those with brain injury induced disabilities both male and female. The incidence rates were 24.0 times (male) and 19.8 times (female) higher in people with intellectual disabilities compared to people without disabilities in recent years. Those with brain injury also showed higher rates of incidence compared to people without disabilities (Male: 5.5 times; Female: 4.6 times, respectively).

### Long-term trends in the age-standardized prevalence of psychotic disorders

**Fig. 2:**
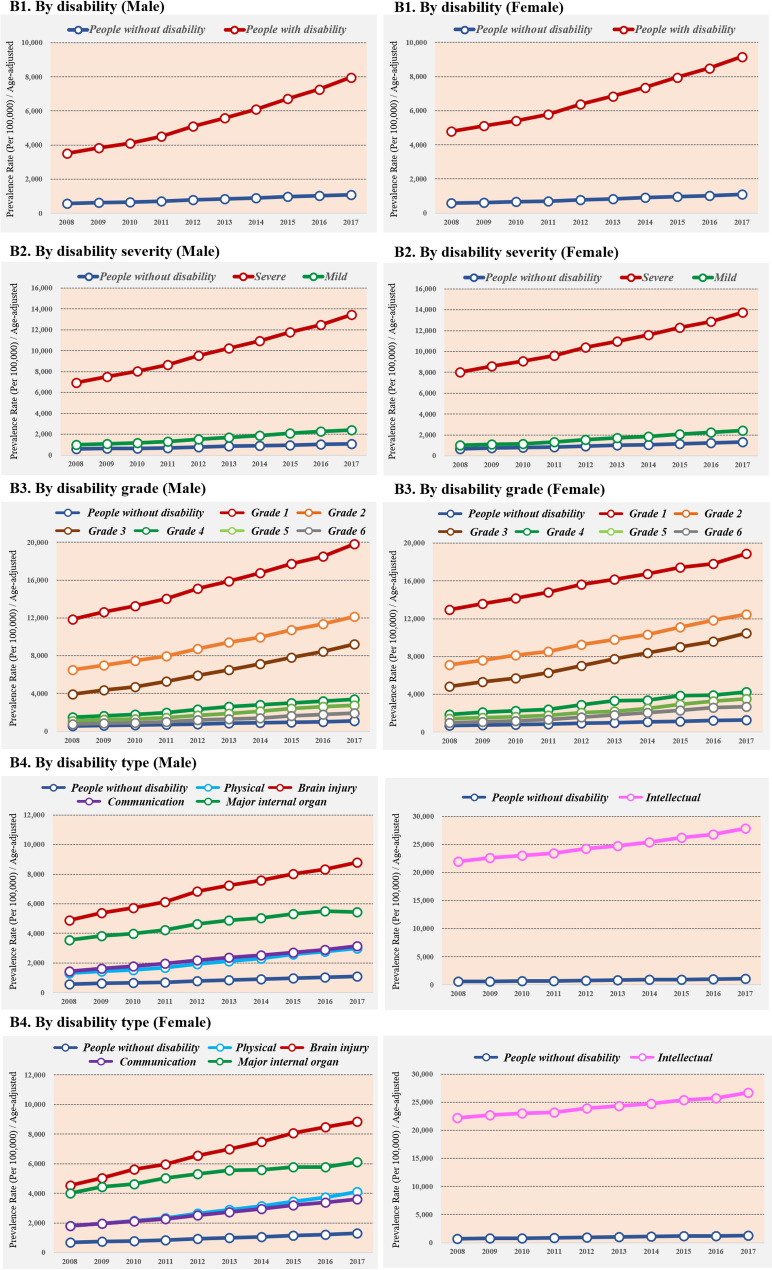
Age-standardized prevalence of psychotic disorders by disability characteristics, 2008 to 2017

The trends in age-standardized prevalence of psychotic disorders between 2008 and 2017 are shown in Fig. 2. The age-standardized prevalence of psychotic disorders consistently increased for both people with and without disabilities in all study years. The prevalence was more than 7.3 times and 7.0 times higher among people with disabilities than among people without disabilities in recent years both male and female; furthermore, it has been found that the gap in the prevalence of psychotic disorders between the people with and without disabilities has widened over time.

The psychotic disorders were more prevalent as the disability severity increased. The age-standardized prevalence of psychotic disorders was highest in people with severe disabilities in all periods, and the prevalence rates were 12.3 times (male) and 10.5 times (female) higher in people with severe disabilities compared to the people without disabilities in recent years. The gap in the prevalence of psychotic disorders between people with severe disabilities the without disabilities sharply widened over time. In particular, for grade 1 with the highest severity, the prevalence of psychotic disorders was 18.2 times (male) and 14.4 times (female) higher than the people without disabilities.

Among the five types of disabilities, the highest prevalence of psychotic disorders was observed among people with intellectual disabilities, followed by those with brain injury induced disabilities both male and female. The prevalence rates were 25.5 times (male) and 20.4 times (female) higher in people with intellectual disabilities compared to people without disabilities in recent years. Those with brain injury also showed higher rates of prevalence compared to people without disabilities (Male: 8.1 times; Female: 6.8 times, respectively).

### Risk of incidence and prevalence of psychotic disorders

The gender-stratified multivariable logistic regression analysis for incidence was sequentially adjusted for age, income level, insurance type, residential area, CCI score, and mental health disease in Table 2. The unadjusted regression analyses of incidence revealed that people with severe disabilities ([Male] OR = 8.557, 95% CI = 8.187–8.943 / [Female] OR = 7.168, 95% CI = 6.823–7.531, respectively), grade 1 ([Male] OR = 13.448, 95% CI = 12.562–14.396 / [Female] OR = 10.043, 95% CI = 9.255–10.899, respectively), and people with intellectual disabilities ([Male] OR = 21.525, 95% CI = 20.433–22.675 / [Female] OR = 16.301, 95% CI = 15.356–17.304, respectively) groups higher psychotic disorders incidences compared to those without a disability both male and female (Model 1). After adjusting age, income level, insurance type, and residential area (Model 3), the odds ratio of incidence was significantly decreased by the largest amount; furthermore, people with severe disabilities, grade 1, and people with intellectual disabilities showed the strongest associations with incidence of psychotic disorders in Model 5 with fully adjusted covariates ([Male] aOR = 2.784, 95% CI = 2.647–2.929; [Female] aOR = 3.143, 95% CI = 2.979–3.135 / [Male] aOR = 4.139, 95% CI = 3.846–4.455; [Female] aOR = 4.463, 95% CI = 4.100-4.859 / [Male] aOR = 5.463, 95% CI = 5.135–5.812; [Female] aOR = 6.845, 95% CI = 6.392–7.330, respectively).

**Table 2:** Factors associated with incidence of psychotic disorder in 2017

**Male**	**Model 1**	**Model 2**	**Model 3**	**Model 4**	**Model 5**
**Disability**					
Yes (vs. No)	8.557(8.187–8.943)	7.845(7.492–8.216)	4.550(4.321–4.792)	4.186(3.974–4.408)	2.784(2.647–2.929)
**By disability severe**	1.950(1.823–2.085)	1.761(1.643–1.888)	1.450(1.352–1.556)	1.353(1.262–1.452)	1.044(0.973–1.120)
Severe (vs. No)	13.448(12.562–14.396)	12.524(11.692–13.415)	6.512(6.042–7.017)	6.001(5.569–6.466)	4.139(3.846–4.455)
Mild (vs. No)	7.543(6.991–8.138)	6.844(6.334–7.394)	4.024(3.712–4.363)	3.582(3.304–3.885)	2.581(2.382–2.797)
Grade 1 (vs. No)	6.670(6.212–7.161)	6.030(5.607–6.484)	3.830(3.552–4.129)	3.604(3.343–3.886)	2.188(2.030–2.358)
Grade 2 (vs. No)	2.654(2.352–2.993)	2.180(1.929–2.465)	1.679(1.485–1.898)	1.560(1.379–1.764)	1.205(1.066–1.363)
Grade 3 (vs. No)	1.995(1.783–2.233)	1.717(1.532–1.926)	1.430(1.275–1.604)	1.319(1.175–1.479)	1.000(0.892–1.122)
Grade 4 (vs. No)	1.589(1.428–1.768)	1.531(1.375–1.706)	1.322(1.186–1.472)	1.248(1.120–1.391)	0.976(0.876–1.088)
Grade 5 (vs. No)	8.557(8.187–8.943)	7.845(7.492–8.216)	4.550(4.321–4.792)	4.186(3.974–4.408)	2.784(2.647–2.929)
Grade 6 (vs. No)	1.950(1.823–2.085)	1.761(1.643–1.888)	1.450(1.352–1.556)	1.353(1.262–1.452)	1.044(0.973–1.120)
**By disability type**					
Physical (vs. No)	1.832(1.699–1.975)	1.628(1.508–1.758)	1.370(1.268–1.48)	1.275(1.180–1.378)	0.981(0.908–1.060)
Brain injury (vs. No)	4.695(4.215–5.231)	3.473(3.110–3.878)	2.720(2.434–3.038)	2.126(1.901–2.377)	1.412(1.263–1.578)
Communication (vs. No)	3.239(2.973–3.528)	2.071(1.893–2.264)	1.790(1.637–1.957)	1.690(1.545–1.848)	1.412(1.292–1.544)
Intellectual (vs. No)	21.525(20.433–22.675)	22.625(21.470-23.843)	10.726(10.062–11.435)	10.896(10.226–11.610)	5.463(5.135–5.812)
Major internal organ (vs. No)	2.043(1.665–2.506)	1.730(1.409–2.123)	1.382(1.126–1.698)	0.962(0.782–1.182)	0.764(0.621–0.939)
**Female**	**Model 1**	**Model 2**	**Model 3**	**Model 4**	**Model 5**
**Disability**					
Yes (vs. No)	3.795(3.640–3.956)	2.818(2.693–2.949)	2.347(2.242–2.457)	2.251(2.150–2.357)	1.805(1.725–1.889)
**By disability severe**					
Severe (vs. No)	7.168(6.823–7.531)	5.678(5.394–5.976)	4.252(4.030–4.486)	4.065(3.852–4.290)	3.143(2.979–3.315)
Mild (vs. No)	1.932(1.804–2.069)	1.325(1.234–1.424)	1.217(1.133–1.307)	1.171(1.090–1.257)	0.958(0.893–1.029)
Grade 1 (vs. No)	10.043(9.255–10.899)	7.906(7.277–8.588)	5.600(5.143–6.097)	5.362(4.924–5.838)	4.463(4.100-4.859)
Grade 2 (vs. No)	6.040(5.535–6.591)	4.830(4.421–5.277)	3.631(3.319–3.973)	3.420(3.125–3.743)	2.846(2.601–3.114)
Grade 3 (vs. No)	6.440(5.956–6.964)	5.076(4.688–5.496)	3.947(3.641–4.278)	3.815(3.519–4.136)	2.660(2.454–2.883)
Grade 4 (vs. No)	2.483(2.214–2.784)	1.466(1.304–1.648)	1.333(1.186–1.498)	1.279(1.138–1.437)	1.068(0.950–1.200)
Grade 5 (vs. No)	1.975(1.773–2.199)	1.314(1.177–1.466)	1.213(1.087–1.354)	1.165(1.044–1.301)	0.950(0.851–1.060)
Grade 6 (vs. No)	1.467(1.289–1.670)	1.192(1.046–1.358)	1.098(0.964–1.252)	1.062(0.932–1.211)	0.859(0.753–0.979)
**By disability type**					
Physical (vs. No)	1.748(1.614–1.893)	1.155(1.064–1.254)	1.090(1.004–1.183)	1.038(0.956–1.127)	0.846(0.779–0.918)
Brain injury (vs. No)	3.070(2.679–3.517)	1.827(1.592–2.097)	1.707(1.487–1.959)	1.502(1.308–1.725)	1.208(1.053–1.387)
Communication (vs. No)	3.521(3.238–3.829)	1.933(1.773–2.108)	1.804(1.654–1.967)	1.760(1.614–1.919)	1.602(1.469–1.747)
Intellectual (vs. No)	16.301(15.356–17.304)	18.646(17.558–19.801)	11.589(10.813–12.421)	11.700(10.919–12.537)	6.845(6.392–7.330)
Major internal organ (vs. No)	1.821(1.430–2.320)	1.583(1.242–2.017)	1.419(1.113–1.808)	1.150(0.902–1.468)	1.013(0.794–1.293)

Model 1: Crude

Model 2: Model 1 + Adjusted for age

Model 3: Model 2 + Adjusted for income, type of insurance, and residence

Model 4: Model 3 + Adjusted for CCI

Model 5: Model 4 + Adjusted for mental disease(depression, anxiety and sleep disorder)

The gender-stratified multivariable logistic regression analysis for prevalence was sequentially adjusted for age, income level, insurance type, residential area, CCI score, and mental health disease in Table 3. The results for prevalence revealed a similar pattern. All disability types and severity were associated with an increased probability for prevalence of psychotic disorders, likewise, people with severe disabilities ([Male] aOR = 6.868, 95% CI = 6.797–6.939; [Female] aOR = 8.308, 95% CI = 8.222–8.394, respectively), and people with intellectual disabilities ([Male] aOR = 45.214, 95% CI = 44.538–45.901; [Female] aOR = 65.091, 95% CI = 64.038–66.161, respectively) have a high odds ratio in Model 5 both male and female.

**Table 3:** Factors associated with prevalence of psychotic disorder in 2017

**Male**	**Model 1**	**Model 2**	**Model 3**	**Model 4**	**Model 5**
**Disability**					
Yes (vs. No)	8.167(8.109–8.225)	7.307(7.251–7.363)	4.344(4.308–4.380)	4.118(4.084–4.153)	3.284(3.255–3.313)
**By disability severe**					
Severe (vs. No)	18.874(18.722–19.026)	16.867(16.726–17.009)	8.584(8.503–8.666)	8.057(7.980–8.134)	6.868(6.797–6.939)
Mild (vs. No)	2.734(2.700-2.769)	2.300(2.270–2.330)	1.752(1.729–1.776)	1.654(1.632–1.676)	1.181(1.164–1.198)
Grade 1 (vs. No)	21.265(20.963–21.572)	19.842(19.557–20.131)	8.467(8.333–8.604)	7.955(7.828–8.083)	7.352(7.221–7.485)
Grade 2 (vs. No)	15.778(15.568–15.990)	13.945(13.755–14.137)	6.894(6.792–6.997)	6.253(6.161–6.347)	5.663(5.569–5.758)
Grade 3 (vs. No)	20.053(19.831–20.277)	17.639(17.438–17.842)	10.102(9.978–10.228)	9.718(9.598–9.839)	7.575(7.469–7.683)
Grade 4 (vs. No)	3.615(3.533–3.699)	2.882(2.815–2.950)	1.946(1.900-1.993)	1.826(1.783–1.871)	1.344(1.310–1.379)
Grade 5 (vs. No)	2.997(2.937–3.059)	2.455(2.405–2.507)	1.885(1.845–1.925)	1.756(1.720–1.794)	1.234(1.207–1.262)
Grade 6 (vs. No)	2.144(2.101–2.188)	1.886(1.848–1.924)	1.531(1.500-1.563)	1.460(1.430–1.490)	1.038(1.015–1.061)
**By disability type**					
Physical (vs. No)	2.686(2.650–2.724)	2.031(2.003–2.060)	1.595(1.573–1.619)	1.490(1.469–1.512)	1.030(1.014–1.046)
Brain injury (vs. No)	9.392(9.223–9.564)	6.180(6.066–6.297)	4.319(4.237–4.403)	3.350(3.285–3.416)	2.209(2.164–2.256)
Communication (vs. No)	3.461(3.398–3.525)	2.041(2.003–2.081)	1.645(1.613–1.677)	1.553(1.524–1.584)	1.243(1.217–1.268)
Intellectual (vs. No)	81.506(80.617–82.405)	92.970(91.933–94.018)	41.481(40.949–42.020)	43.408(42.852–43.972)	45.214(44.538–45.901)
Major internal organ (vs. No)	4.934(4.790–5.083)	3.588(3.482–3.698)	2.539(2.462–2.617)	1.746(1.693–1.801)	1.317(1.274–1.362)
**Female**	**Model 1**	**Model 2**	**Model 3**	**Model 4**	**Model 5**
**Disability**					
Yes (vs. No)	7.284(7.232–7.337)	5.475(5.432–5.518)	4.153(4.119–4.186)	3.965(3.933–3.997)	3.245(3.218–3.273)
**By disability severe**					
Severe (vs. No)	17.295(17.148–17.444)	13.884(13.762–14.008)	9.310(9.222–9.398)	8.855(8.771–8.939)	8.308(8.222–8.394)
Mild (vs. No)	2.723(2.689–2.757)	1.813(1.789–1.837)	1.583(1.563–1.604)	1.512(1.492–1.532)	1.146(1.131–1.162)
Grade 1 (vs. No)	15.245(14.991–15.504)	12.529(12.316–12.746)	7.566(7.432–7.703)	7.196(7.069–7.326)	7.436(7.290–7.584)
Grade 2 (vs. No)	13.658(13.460-13.859)	10.914(10.753–11.079)	7.250(7.139–7.364)	6.734(6.630–6.840)	6.743(6.628–6.860)
Grade 3 (vs. No)	22.005(21.745–22.269)	17.507(17.294–17.722)	12.446(12.288–12.605)	12.049(11.896–12.203)	10.200(10.055–10.347)
Grade 4 (vs. No)	3.229(3.160–3.300)	1.952(1.909–1.996)	1.662(1.625-1.700)	1.579(1.544–1.615)	1.251(1.222–1.280)
Grade 5 (vs. No)	2.814(2.759–2.869)	1.837(1.801–1.874)	1.616(1.584–1.649)	1.537(1.506–1.568)	1.156(1.132–1.180)
Grade 6 (vs. No)	2.246(2.196–2.296)	1.666(1.629–1.704)	1.470(1.436–1.504)	1.410(1.378–1.443)	1.040(1.016–1.065)
**By disability type**					
Physical (vs. No)	2.642(2.605–2.679)	1.541(1.519–1.563)	1.424(1.404–1.445)	1.336(1.316–1.355)	0.994(0.980–1.009)
Brain injury (vs. No)	7.408(7.259–7.559)	4.085(4.001–4.171)	3.682(3.606–3.760)	3.100(3.035–3.166)	2.517(2.463–2.573)
Communication (vs. No)	3.323(3.262–3.386)	1.719(1.686–1.752)	1.566(1.536–1.596)	1.513(1.484–1.543)	1.347(1.321–1.374)
Intellectual (vs. No)	76.447(75.514–77.393)	92.248(91.092–93.419)	54.523(53.767–55.289)	56.384(55.602–57.178)	65.091(64.038–66.161)
Major internal organ (vs. No)	4.248(4.103–4.399)	3.178(3.068–3.292)	2.695(2.602–2.792)	2.042(1.970–2.116)	1.801(1.735–1.870)

Model 1: Crude

Model 2: Model 1 + Adjusted for age

Model 3: Model 2 + Adjusted for income, type of insurance, and residence

Model 4: Model 3 + Adjusted for CCI

Model 5: Model 4 + Adjusted for mental disease(depression, anxiety and sleep disorder)

## Discussion

To the best of our knowledge, this is the first study to comprehensively analyze potential disparities in the incidence and prevalence of psychotic disorders according to disability status. The strengths of this study included the large sample sizes and long-term analysis trends. This is the first study to conduct a comprehensive examination of the long-term trends in the incidence and prevalence of psychotic disorders, and association of disabled status. Furthermore, our dataset covers the entire population of South Korea, extended over a 10-year period from 2008 to 2017. Moreover, the adjustment for risk factors such as age, residential area, income level, mental health, and comorbidities represents a robust aspect of the current findings.

A notable discovery from our study is the marked incidence and prevalence of psychotic disorders in the disabled population, especially in people with severe disabilities. The incidence and prevalence were 3.04 and 7.64 times higher among people with severe disabilities compared to people without disabilities in 2017, respectively. This suggests that when people with disabilities develop a psychotic disorder, they are less likely to be treated timeously and are affected by the disease for a longer period of time than people without disabilities. One important reason for these finding may be the low socioeconomic status of people with disabilities. Disabilities are directly associated with a low employment rate and low economic activity [15, 20–22]. The relationship between low socioeconomic status and psychotic disorders has also been reported in previous studies. Among all severe mental disorders, schizophrenia exhibits the most pronounced association with poverty [23]. According to a recent France study, more than 8 out of 10 schizophrenia patients live below the poverty threshold [23]and a study conducted within the Danish population has also revealed that a low parental income was associated with an increased risk of schizophrenia onset in their offspring [24]. In this study, regression analyses revealed that adjusting for socioeconomic status such as level of income, type of insurance, and residence (Model 3) significantly reduced the odds ratios in incidence and prevalence. Schizophrenia presents with several clinical factors (including delusions, negative symptoms, and poor insight into illness) as well as social stigma, which hampers employment opportunities [23]. People with disabilities are more likely to join the double stigma of ‘disability and ‘schizophrenia’. Although stigma is not a direct mental health outcome, it can influence treatment adherence and overall well-being among individuals with schizophrenia; eventually these lead to longer periods of suffering compared to people without disabilities [25]. The government must increase effort to fostering social inclusion for disabled people with schizophrenia and narrow the gap between people with and without disabilities. Social inclusion should be understood not only as access to services but as full participation in society-including relationships, employment, education, and community life. To support this, professionals and community members can help individuals identify personal goals and connect with resources that address barriers in their daily lives [26]. These efforts can promote more integrated, meaningful social participation for people with schizophrenia and should be reflected in mental health and disability policy.

One possible explanation for the higher incidence and prevalence of psychotic disorders among people with disabilities may involve factors such as poor mental health or social isolation, as suggested by previous studies. Although we could not establish the reasons for the high incidence and prevalence of psychotic disorders in people with disabilities, the results obtained are similar to those of previous studies. The pathways between disability and psychotic disorders are unclear but may include the mediating roles of social support or social deafferentation, as well as mental health aspects such as depression, sleep deprivation, or psychological distress [27–30]. Schizophrenia is often associated with social isolation, and multiple studies suggest that social isolation tends to precede the onset of psychotic symptoms [31–33]. Epidemiologically, an early study by Faris and Dunham found that living conditions fostering social isolation in Chicago were associated with an increased incidence of schizophrenia [34]. Moreover, among the cases surveyed who could recall the onset of auditory/verbal hallucinations, increasing social isolation prior to hallucination onset was reported at a rate of 73% [35]. People with disabilities usually face various problems leading to social isolation. Mental health conditions are associated with feelings of loneliness, and social isolation, which are closely linked to depression. Previous studies have shown that depression occurs more frequently in adults with disabilities than in people without disabilities [11]. Furthermore, in model 5 of the adjusted analysis for prevalence and incidence, regression analyses revealed that adjusting for mental health, such as depression, sleep disorder, or anxiety, reduced the odds ratios significantly in incidence and prevalence. Ultimately, social isolation and poor mental health may be associated with a higher likelihood of schizophrenia. One study demonstrated for the first time that psychosocial interventions may effectively improve symptomatic outcomes even in the absence of medications [36]. It seems necessary to discuss from a public health perspective what efforts can be made to prevent and manage schizophrenia in vulnerable groups such as the disabled. For example, preventive approaches to hypertension or obesity do not focus on identifying individuals carrying biological markers; rather they encourage members of the general population to regularly exercise and reduce their calorie intake. A similar approach should be adopted for psychosis [37]. A potentially more effective approach may be to target factors that are associated with a higher likelihood of developing schizophrenia. For example, locating nearby support groups, assisting with daily medication adherence, encouraging therapy, or seeking assistance for stress management, are important for people with disabilities to manage stressful life events and prevent triggers for the initiation of schizophrenia. Where possible, people with disabilities that are at risk should have a network they can trust to communicate with and avoid isolation.

Another key finding of this study was significant differences in incidence and prevalence of psychotic disorders according to disability type. The age-adjusted incidence and prevalence rates were higher in people with intellectual disabilities and brain injury. The results of previous studies were also similar to our study. Intellectual disabilities (ID) have been associated with a higher prevalence of psychiatric problems. More recent research has revealed that individuals with ID at all levels, even individuals with profound ID who do not communicate verbally, are at a higher risk of developing psychiatric conditions than people within the normal IQ range [12, 13]. According to Morgan’s research, 31.7% of people with intellectual disabilities had psychiatric disorders, and 3.7–5.2% of those with intellectual disabilities had co-occurring schizophrenia [38]. Moreover, people with both ID and autism concurrently are more vulnerable to mental health problems, than people with ID only [39–41]. In addition, psychiatric illness was found to be more prevalent among people with brain injury. The pathways between brain impairment and psychotic disorders are unclear, but may include the mediating roles of neuropsychological impairment [42]. Disability with brain injury means that the person sustained damage to the brain after birth, caused by an accident or trauma, a stroke, disease (ex: brain tumor) or infection. Mental disorders related traumatic brain injury (TBI) have been hypothesized in literature prior to 1969 as the etiology of schizophrenia [43]and recent evidence suggests that traumatic brain injury may be one such trigger of schizophrenia. According to previous research, traumatic brain injuries were significantly higher for individuals with a diagnosis of schizophrenia, bipolar disorder, and depression than for those with no mental illness [44].Traumatic brain injury (TBI) is associated with significant adverse mental health outcomes in up to one-third of survivors [45]thus increasing the risk of psychosis, perhaps doubling it [46]. While these findings do indicate a significant association between TBI and schizophrenia, the causal relationship remains unclear. In light of this relationship, public health policies should be aware that individuals with ID or brain injury may need specialized monitoring and intervention to avoid the development of psychotic disorders such as schizophrenia. However, the epidemiology of people with intellectual disabilities or brain injury co-occurring with schizophrenia and other psychiatric illnesses is poorly understood. Future research should analyze the mechanisms connecting the type of disability to a higher incidence or prevalence of psychotic disorders.

There were some limitations to the present study. First, because it was a serial cross-sectional study using claim database, causality between disability and psychotic experience could not be inferred. Second, it was not possible to collect data on all factors that could be associated with outcomes such as clinical data (ex: obstetric complications, infections…), genes (ex: family medical history…), environmental factors (ex: winter or spring birth, or migration…), or psychosocial factors (ex: heavy use of cannabis…). Since we only evaluated the risk of psychotic disorders due to disability without considering the factors for typical prodromal symptoms of psychotic disorders, future studies should clarify the causation relationships between psychotic disorders and disabilities by further investigating other factors that affect the incidence or prevalence of psychotic disorders. Third, we lacked important demographic variables that may influence psychotic disorders such as marital status, education level, employment status, family history of mental illness, and environmental exposures. Fourth, although gender-stratified analyses were conducted, we could not examine interaction effects between gender and other sociodemographic factors, which may underlie gender-specific disparities in mental health among people with disabilities. Additionally, theoretical considerations related to gender-specific medicine were not incorporated and should be addressed in future research. However, we believe these results provide important insight for strategies to prevent the development of schizophrenia.

## Supplementary Information

Below is the link to the electronic supplementary material.

Supplementary Material 1

Supplementary Material 2

## Data Availability

Data sharing is not applicable to this article due to technical or time limitations.
