# Supplementary material for: Disparities in the incidence and prevalence of psychotic disorders among people with and without disabilities in south Korea: A national database study
Source: Arch Public Health. 2025 Aug 4;83:205. doi: 10.1186/s13690-025-01691-4 (PMC12323167; doi:10.1186/s13690-025-01691-4)
Supplement: Supplementary file 1 — Supplementary Material 1 [file 13690_2025_1691_MOESM1_ESM.docx]

**Supplement Table 1. Categorization of type of disabilities**

| **Category** | **Type of disability** |
| --- | --- |
| 1. Physical | amputation, motor disturbance, joint disability, disfigurements or deformities, spinal cord injury |
| 1. Brain injury | brain impairment |
| 1. Communication | sight, hearing or speech problems |
| 1. Intellectual | difficulty learning or understanding things |
| 1. Major internal organ | functional impairment of internal organ diseases such as kidney, heart, liver, lung, intestine |
|  | |

**Supplement Table 2. Incidence rate of psycho disorders during 2008~2017 (Male)**

| **Year** | **2008** | **2009** | **2010** | **2011** | **2012** | **2013** | **2014** | **2015** | **2016** | **2017** |
| --- | --- | --- | --- | --- | --- | --- | --- | --- | --- | --- |
| **By disability** |  |  |  |  |  |  |  |  |  |  |
| People without disability |  |  |  |  |  |  |  |  |  |  |
| Incidence rate (crude) | 69.8 | 64.1 | 50.7 | 52.7 | 57.8 | 50.9 | 50.6 | 53.7 | 53.8 | 51.7 |
| Incidence rate (age-adjusted) | 72.4 | 66.3 | 52.6 | 54.5 | 58.9 | 51.9 | 51.4 | 54.1 | 54.1 | 51.8 |
| People with disability |  |  |  |  |  |  |  |  |  |  |
| Incidence rate (crude) | 254.3 | 231.4 | 184.6 | 195.7 | 248.4 | 178.3 | 183.5 | 202.0 | 185.0 | 211.4 |
| Incidence rate (age-adjusted) | 351.6 | 333.8 | 273.6 | 278.1 | 390.4 | 280.2 | 297.0 | 339.8 | 298.3 | 388.3 |
| **By severity** |  |  |  |  |  |  |  |  |  |  |
| Severe (Grade 1-3) |  |  |  |  |  |  |  |  |  |  |
| Incidence rate (crude) | 454.5 | 419.1 | 326.2 | 359.6 | 463.6 | 322.7 | 333.3 | 371.3 | 323.6 | 411.7 |
| Incidence rate (age-adjusted) | 640.7 | 600.5 | 480.2 | 498.4 | 674.3 | 470.3 | 499.3 | 550.4 | 472.1 | 636.4 |
| Mild (Grade 4-6) |  |  |  |  |  |  |  |  |  |  |
| Incidence rate (crude) | 126.2 | 113.7 | 98.0 | 98.4 | 112.2 | 95.6 | 98.5 | 107.0 | 108.1 | 101.8 |
| Incidence rate (age-adjusted) | 117.6 | 106.1 | 90.5 | 91.5 | 98.2 | 85.8 | 82.0 | 104.1 | 91.1 | 85.4 |
| Grade 1 |  |  |  |  |  |  |  |  |  |  |
| Incidence rate (crude) | 715.3 | 646.9 | 456.8 | 538.0 | 632.9 | 499.2 | 529.8 | 603.0 | 493.7 | 689.6 |
| Incidence rate (age-adjusted) | 872.2 | 842.8 | 579.6 | 655.3 | 807.1 | 621.3 | 673.8 | 769.7 | 606.9 | 918.2 |
| Grade 2 |  |  |  |  |  |  |  |  |  |  |
| Incidence rate (crude) | 452.5 | 421.1 | 327.7 | 345.6 | 423.0 | 303.7 | 302.2 | 336.5 | 292.3 | 371.2 |
| Incidence rate (age-adjusted) | 632.4 | 558.9 | 485.5 | 475.1 | 641.1 | 430.5 | 450.4 | 493.3 | 437.8 | 576.1 |
| Grade 3 |  |  |  |  |  |  |  |  |  |  |
| Incidence rate (crude) | 303.9 | 284.0 | 247.9 | 265.4 | 401.6 | 233.1 | 240.9 | 258.7 | 245.2 | 274.8 |
| Incidence rate (age-adjusted) | 480.3 | 438.0 | 389.2 | 395.7 | 581.9 | 367.0 | 378.8 | 380.4 | 379.0 | 410.8 |
| Grade 4 |  |  |  |  |  |  |  |  |  |  |
| Incidence rate (crude) | 160.3 | 138.2 | 118.1 | 145.1 | 146.4 | 146.3 | 123.7 | 135.3 | 139.8 | 138.5 |
| Incidence rate (age-adjusted) | 168.5 | 148.4 | 127.7 | 147.4 | 139.4 | 156.1 | 116.4 | 138.4 | 127.7 | 130.0 |
| Grade 5 |  |  |  |  |  |  |  |  |  |  |
| Incidence rate (crude) | 125.0 | 132.3 | 99.3 | 97.5 | 122.4 | 95.5 | 105.9 | 107.4 | 105.4 | 104.2 |
| Incidence rate (age-adjusted) | 115.5 | 120.3 | 76.4 | 89.8 | 108.3 | 79.1 | 89.3 | 118.3 | 100.9 | 79.3 |
| Grade 6 |  |  |  |  |  |  |  |  |  |  |
| Incidence rate (crude) | 109.9 | 88.1 | 87.1 | 75.9 | 88.3 | 71.4 | 81.5 | 93.7 | 95.4 | 83.0 |
| Incidence rate (age-adjusted) | 102.2 | 84.1 | 85.4 | 74.0 | 78.0 | 66.7 | 68.9 | 90.2 | 75.9 | 75.7 |
| **By disability type** |  |  |  |  |  |  |  |  |  |  |
| Physical |  |  |  |  |  |  |  |  |  |  |
| Incidence rate (crude) | 136.2 | 126.4 | 94.8 | 98.9 | 110.3 | 91.5 | 100.5 | 107.3 | 97.1 | 95.6 |
| Incidence rate (age-adjusted) | 146.5 | 135.3 | 105.9 | 110.2 | 127.7 | 104.0 | 119.0 | 125.1 | 118.2 | 129.9 |
| Brain injury |  |  |  |  |  |  |  |  |  |  |
| Incidence rate (crude) | 297.4 | 311.1 | 239.5 | 263.3 | 308.3 | 251.1 | 206.9 | 234.8 | 237.0 | 244.8 |
| Incidence rate (age-adjusted) | 425.1 | 344.9 | 305.6 | 330.5 | 388.1 | 251.5 | 221.4 | 291.8 | 207.0 | 287.5 |
| Communication |  |  |  |  |  |  |  |  |  |  |
| Incidence rate (crude) | 187.7 | 143.7 | 149.3 | 166.1 | 172.3 | 151.7 | 162.1 | 172.4 | 167.6 | 168.9 |
| Incidence rate (age-adjusted) | 175.6 | 149.5 | 147.8 | 143.7 | 167.7 | 144.2 | 138.5 | 151.0 | 134.1 | 156.2 |
| Intellectual |  |  |  |  |  |  |  |  |  |  |
| Incidence rate (crude) | 1,736.1 | 1,526.9 | 1,211.9 | 1,207.7 | 1,276.4 | 990.4 | 1,009.8 | 1,094.1 | 918.9 | 1,254.3 |
| Incidence rate (age-adjusted) | 1,721.1 | 1,497.6 | 1,193.3 | 1,214.0 | 1,291.3 | 949.2 | 971.9 | 1,061.2 | 882.9 | 1,241.7 |
| Major internal organ |  |  |  |  |  |  |  |  |  |  |
| Incidence rate (crude) | 271.4 | 295.0 | 162.1 | 170.8 | 159.7 | 134.6 | 104.4 | 126.4 | 109.3 | 106.6 |
| Incidence rate (age-adjusted) | 338.4 | 379.2 | 252.7 | 175.9 | 211.0 | 172.1 | 132.9 | 129.9 | 186.9 | 120.6 |

**Supplement Table 3. Incidence rate of psycho disorders during 2008~2017 (Female)**

| **Year** | **2008** | **2009** | **2010** | **2011** | **2012** | **2013** | **2014** | **2015** | **2016** | **2017** |
| --- | --- | --- | --- | --- | --- | --- | --- | --- | --- | --- |
| **By disability** |  |  |  |  |  |  |  |  |  |  |
| People without disability |  |  |  |  |  |  |  |  |  |  |
| Incidence rate (crude) | 103.8 | 92.8 | 56.1 | 59.0 | 67.1 | 60.3 | 59.9 | 64.7 | 64.5 | 65.1 |
| Incidence rate (age-adjusted) | 103.5 | 92.2 | 54.3 | 55.9 | 63.1 | 56.6 | 56.1 | 60.1 | 60.1 | 60.6 |
| People with disability |  |  |  |  |  |  |  |  |  |  |
| Incidence rate (crude) | 245.9 | 219.5 | 184.8 | 174.3 | 206.4 | 176.2 | 182.1 | 194.9 | 179.0 | 222.8 |
| Incidence rate (age-adjusted) | 444.5 | 395.3 | 375.3 | 336.6 | 399.3 | 318.3 | 347.4 | 355.0 | 321.5 | 418.3 |
| **By severity** |  |  |  |  |  |  |  |  |  |  |
| Severe (Grade 1-3) |  |  |  |  |  |  |  |  |  |  |
| Incidence rate (crude) | 452.3 | 405.2 | 366.8 | 324.0 | 383.0 | 320.6 | 333.2 | 337.9 | 309.0 | 419.1 |
| Incidence rate (age-adjusted) | 670.8 | 602.8 | 586.3 | 509.2 | 594.7 | 477.8 | 512.2 | 517.6 | 457.0 | 635.8 |
| Mild (Grade 4-6) |  |  |  |  |  |  |  |  |  |  |
| Incidence rate (crude) | 119.8 | 113.8 | 84.8 | 94.0 | 113.8 | 101.7 | 105.1 | 122.8 | 114.4 | 126.8 |
| Incidence rate (age-adjusted) | 163.7 | 139.9 | 107.9 | 97.3 | 123.6 | 98.9 | 103.9 | 101.5 | 108.8 | 80.2 |
| Grade 1 |  |  |  |  |  |  |  |  |  |  |
| Incidence rate (crude) | 644.1 | 516.3 | 501.2 | 431.9 | 511.7 | 442.5 | 450.7 | 447.7 | 389.3 | 641.1 |
| Incidence rate (age-adjusted) | 891.1 | 689.0 | 712.7 | 599.0 | 678.7 | 590.9 | 618.5 | 613.5 | 490.0 | 861.1 |
| Grade 2 |  |  |  |  |  |  |  |  |  |  |
| Incidence rate (crude) | 431.2 | 415.6 | 359.3 | 308.4 | 373.5 | 321.4 | 318.1 | 317.5 | 308.2 | 377.0 |
| Incidence rate (age-adjusted) | 634.6 | 636.2 | 555.3 | 451.6 | 579.0 | 444.8 | 475.3 | 465.1 | 445.4 | 542.4 |
| Grade 3 |  |  |  |  |  |  |  |  |  |  |
| Incidence rate (crude) | 328.8 | 312.4 | 276.0 | 262.0 | 301.2 | 234.4 | 265.2 | 279.5 | 252.5 | 302.2 |
| Incidence rate (age-adjusted) | 517.2 | 492.0 | 505.4 | 507.3 | 544.3 | 401.0 | 444.6 | 479.7 | 450.8 | 519.4 |
| Grade 4 |  |  |  |  |  |  |  |  |  |  |
| Incidence rate (crude) | 132.4 | 122.2 | 95.8 | 108.0 | 133.6 | 122.8 | 125.8 | 144.9 | 145.3 | 162.9 |
| Incidence rate (age-adjusted) | 192.4 | 180.2 | 177.3 | 92.2 | 227.8 | 135.9 | 126.5 | 159.6 | 156.5 | 130.7 |
| Grade 5 |  |  |  |  |  |  |  |  |  |  |
| Incidence rate (crude) | 118.6 | 109.1 | 90.0 | 88.0 | 111.0 | 97.2 | 104.9 | 128.7 | 121.6 | 129.6 |
| Incidence rate (age-adjusted) | 186.0 | 138.8 | 107.8 | 91.2 | 79.4 | 103.5 | 98.6 | 95.5 | 123.5 | 70.4 |
| Grade 6 |  |  |  |  |  |  |  |  |  |  |
| Incidence rate (crude) | 110.7 | 112.2 | 68.5 | 88.6 | 99.8 | 89.3 | 88.5 | 99.1 | 82.9 | 96.3 |
| Incidence rate (age-adjusted) | 136.6 | 123.6 | 76.6 | 104.9 | 105.6 | 83.0 | 97.0 | 79.2 | 78.0 | 66.1 |
| **By disability type** |  |  |  |  |  |  |  |  |  |  |
| Physical |  |  |  |  |  |  |  |  |  |  |
| Incidence rate (crude) | 124.0 | 116.1 | 86.7 | 90.3 | 105.3 | 93.1 | 99.5 | 104.5 | 95.9 | 114.7 |
| Incidence rate (age-adjusted) | 196.1 | 161.8 | 144.5 | 135.8 | 168.7 | 120.7 | 151.4 | 146.0 | 118.0 | 154.2 |
| Brain injury |  |  |  |  |  |  |  |  |  |  |
| Incidence rate (crude) | 277.7 | 222.4 | 216.4 | 199.3 | 245.3 | 194.1 | 190.8 | 201.3 | 197.9 | 201.3 |
| Incidence rate (age-adjusted) | 440.7 | 378.8 | 366.3 | 206.7 | 257.3 | 259.8 | 205.4 | 225.5 | 281.6 | 276.2 |
| Communication |  |  |  |  |  |  |  |  |  |  |
| Incidence rate (crude) | 189.6 | 178.9 | 167.0 | 166.4 | 202.5 | 184.9 | 183.2 | 228.2 | 204.2 | 230.8 |
| Incidence rate (age-adjusted) | 189.8 | 181.7 | 193.4 | 153.2 | 177.4 | 167.4 | 160.4 | 174.3 | 161.0 | 144.4 |
| Intellectual |  |  |  |  |  |  |  |  |  |  |
| Incidence rate (crude) | 1,626.4 | 1,430.8 | 1,316.5 | 1,103.3 | 1,231.6 | 947.7 | 987.8 | 956.3 | 816.6 | 1,202.5 |
| Incidence rate (age-adjusted) | 1,583.4 | 1,457.9 | 1,285.2 | 1,083.2 | 1,224.9 | 951.2 | 969.5 | 946.1 | 802.2 | 1,198.2 |
| Major internal organ |  |  |  |  |  |  |  |  |  |  |
| Incidence rate (crude) | 298.8 | 333.4 | 161.7 | 135.2 | 162.9 | 157.0 | 128.8 | 102.3 | 125.0 | 119.3 |
| Incidence rate (age-adjusted) | 360.5 | 389.4 | 201.2 | 209.5 | 244.1 | 229.9 | 193.1 | 173.7 | 102.2 | 111.0 |

**Supplement Table 4. Prevalence rate of psycho disorders during 2008~2017 (Male)**

| **Year** | **2008** | **2009** | **2010** | **2011** | **2012** | **2013** | **2014** | **2015** | **2016** | **2017** |
| --- | --- | --- | --- | --- | --- | --- | --- | --- | --- | --- |
| **By disability** |  |  |  |  |  |  |  |  |  |  |
| People without disability |  |  |  |  |  |  |  |  |  |  |
| Prevalence rate (crude) | 558.2 | 598.0 | 634.2 | 684.2 | 766.6 | 835.2 | 900.7 | 976.9 | 1,049.5 | 1,127.3 |
| Prevalence rate (age-adjusted) | 582.9 | 620.9 | 655.0 | 702.5 | 780.4 | 840.6 | 897.1 | 963.4 | 1,025.9 | 1,091.4 |
| People with disability |  |  |  |  |  |  |  |  |  |  |
| Prevalence rate (crude) | 2,873.0 | 3,115.9 | 3,310.6 | 3,586.0 | 4,007.2 | 4,336.1 | 4,666.3 | 5,079.6 | 5,389.3 | 5,725.1 |
| Prevalence rate (age-adjusted) | 3,520.1 | 3,835.6 | 4,108.1 | 4,494.7 | 5,093.0 | 5,583.6 | 6,102.6 | 6,717.9 | 7,285.4 | 7,986.1 |
| **By severity** |  |  |  |  |  |  |  |  |  |  |
| Severe (Grade 1-3) |  |  |  |  |  |  |  |  |  |  |
| Prevalence rate (crude) | 5,578.3 | 6,061.3 | 6,463.5 | 6,978.1 | 7,677.1 | 8,225.2 | 8,774.0 | 9,478.0 | 9,996.8 | 10,609.4 |
| Prevalence rate (age-adjusted) | 6,927.9 | 7,519.1 | 8,018.8 | 8,644.8 | 9,522.7 | 10,211.8 | 10,921.4 | 11,772.8 | 12,475.5 | 13,451.5 |
| Mild (Grade 4-6) |  |  |  |  |  |  |  |  |  |  |
| Prevalence rate (crude) | 1,141.0 | 1,268.0 | 1,380.7 | 1,570.7 | 1,873.5 | 2,109.5 | 2,335.5 | 2,610.3 | 2,833.2 | 3,051.0 |
| Prevalence rate (age-adjusted) | 999.6 | 1,092.2 | 1,165.3 | 1,303.7 | 1,533.4 | 1,700.6 | 1,856.9 | 2,085.7 | 2,252.9 | 2,409.0 |
| Grade 1 |  |  |  |  |  |  |  |  |  |  |
| Prevalence rate (crude) | 10,524.0 | 11,273.7 | 11,849.3 | 12,612.7 | 13,622.2 | 14,392.5 | 15,213.5 | 16,134.5 | 16,795.8 | 17,827.1 |
| Prevalence rate (age-adjusted) | 11,858.1 | 12,631.5 | 13,265.6 | 14,033.6 | 15,104.3 | 15,896.7 | 16,753.3 | 17,750.9 | 18,511.8 | 19,841.1 |
| Grade 2 |  |  |  |  |  |  |  |  |  |  |
| Prevalence rate (crude) | 5,320.1 | 5,784.0 | 6,181.0 | 6,624.9 | 7,269.8 | 7,814.1 | 8,251.8 | 8,876.2 | 9,324.5 | 9,788.8 |
| Prevalence rate (age-adjusted) | 6,479.4 | 6,995.7 | 7,450.3 | 7,938.0 | 8,756.9 | 9,413.0 | 9,964.7 | 10,744.3 | 11,368.3 | 12,136.7 |
| Grade 3 |  |  |  |  |  |  |  |  |  |  |
| Prevalence rate (crude) | 2,902.6 | 3,227.4 | 3,511.7 | 3,936.2 | 4,489.9 | 4,890.5 | 5,346.0 | 5,918.2 | 6,389.4 | 6,865.0 |
| Prevalence rate (age-adjusted) | 3,916.0 | 4,379.5 | 4,713.3 | 5,269.1 | 5,926.0 | 6,487.3 | 7,130.8 | 7,792.7 | 8,444.6 | 9,200.3 |
| Grade 4 |  |  |  |  |  |  |  |  |  |  |
| Prevalence rate (crude) | 1,534.2 | 1,688.3 | 1,863.1 | 2,160.2 | 2,560.6 | 2,824.8 | 3,099.2 | 3,439.1 | 3,721.9 | 3,995.2 |
| Prevalence rate (age-adjusted) | 1,486.1 | 1,619.9 | 1,766.7 | 1,994.4 | 2,313.9 | 2,595.6 | 2,803.6 | 3,000.0 | 3,181.9 | 3,403.3 |
| Grade 5 |  |  |  |  |  |  |  |  |  |  |
| Prevalence rate (crude) | 1,261.9 | 1,415.4 | 1,514.2 | 1,720.4 | 2,076.7 | 2,354.0 | 2,616.3 | 2,898.0 | 3,137.7 | 3,334.2 |
| Prevalence rate (age-adjusted) | 1,089.3 | 1,226.2 | 1,291.2 | 1,458.6 | 1,700.3 | 1,874.9 | 2,113.1 | 2,412.1 | 2,610.2 | 2,767.7 |
| Grade 6 |  |  |  |  |  |  |  |  |  |  |
| Prevalence rate (crude) | 858.1 | 954.0 | 1,047.2 | 1,171.6 | 1,393.9 | 1,596.2 | 1,782.0 | 2,029.0 | 2,210.4 | 2,408.5 |
| Prevalence rate (age-adjusted) | 769.8 | 838.0 | 900.9 | 990.8 | 1,179.8 | 1,314.8 | 1,418.1 | 1,626.3 | 1,772.7 | 1,915.5 |
| **By disability type** |  |  |  |  |  |  |  |  |  |  |
| Physical |  |  |  |  |  |  |  |  |  |  |
| Prevalence rate (crude) | 1,290.0 | 1,428.9 | 1,538.2 | 1,694.5 | 1,937.4 | 2,134.6 | 2,345.5 | 2,599.4 | 2,791.5 | 2,998.3 |
| Prevalence rate (age-adjusted) | 1,310.5 | 1,432.2 | 1,519.8 | 1,685.8 | 1,929.1 | 2,108.1 | 2,320.0 | 2,590.4 | 2,785.7 | 3,002.3 |
| Brain injury |  |  |  |  |  |  |  |  |  |  |
| Prevalence rate (crude) | 5,089.9 | 5,655.9 | 6,105.0 | 6,524.8 | 7,270.8 | 7,795.0 | 8,277.8 | 8,891.8 | 9,329.8 | 9,755.7 |
| Prevalence rate (age-adjusted) | 4,883.1 | 5,393.4 | 5,718.2 | 6,110.6 | 6,839.1 | 7,243.1 | 7,567.8 | 8,027.4 | 8,338.7 | 8,791.9 |
| Communication |  |  |  |  |  |  |  |  |  |  |
| Prevalence rate (crude) | 1,561.3 | 1,750.2 | 1,915.4 | 2,184.5 | 2,546.1 | 2,817.3 | 3,070.5 | 3,387.1 | 3,622.9 | 3,829.7 |
| Prevalence rate (age-adjusted) | 1,452.8 | 1,623.7 | 1,766.9 | 1,962.2 | 2,188.6 | 2,368.0 | 2,518.2 | 2,707.4 | 2,899.6 | 3,146.0 |
| Intellectual |  |  |  |  |  |  |  |  |  |  |
| Prevalence rate (crude) | 21,257.6 | 21,900.8 | 22,248.7 | 22,616.0 | 23,397.4 | 23,902.6 | 24,513.9 | 25,279.9 | 25,874.2 | 26,922.7 |
| Prevalence rate (age-adjusted) | 21,940.4 | 22,636.4 | 23,018.3 | 23,434.7 | 24,209.3 | 24,713.3 | 25,364.6 | 26,189.0 | 26,787.1 | 27,820.2 |
| Major internal organ |  |  |  |  |  |  |  |  |  |  |
| Prevalence rate (crude) | 3,208.9 | 3,600.3 | 3,801.7 | 4,154.9 | 4,572.6 | 4,797.3 | 4,835.8 | 5,149.8 | 5,261.4 | 5,374.0 |
| Prevalence rate (age-adjusted) | 3,532.9 | 3,813.3 | 3,985.8 | 4,229.5 | 4,629.9 | 4,881.8 | 5,024.4 | 5,326.9 | 5,489.2 | 5,441.5 |

**Supplement Table 5. Prevalence rate of psycho disorders during 2008~2017 (Female)**

| **Year** | **2008** | **2009** | **2010** | **2011** | **2012** | **2013** | **2014** | **2015** | **2016** | **2017** |
| --- | --- | --- | --- | --- | --- | --- | --- | --- | --- | --- |
| **By disability** |  |  |  |  |  |  |  |  |  |  |
| People without disability |  |  |  |  |  |  |  |  |  |  |
| Prevalence rate (crude) | 707.8 | 782.7 | 834.2 | 901.1 | 1,025.1 | 1,105.6 | 1,195.9 | 1,303.6 | 1,399.2 | 1,510.8 |
| Prevalence rate (age-adjusted) | 684.5 | 750.0 | 790.6 | 842.3 | 931.9 | 1,002.9 | 1,071.0 | 1,152.0 | 1,224.0 | 1,309.3 |
| People with disability |  |  |  |  |  |  |  |  |  |  |
| Prevalence rate (crude) | 3,167.4 | 3,376.3 | 3,592.7 | 3,877.6 | 4,316.2 | 4,726.6 | 5,126.3 | 5,632.2 | 5,995.5 | 6,417.8 |
| Prevalence rate (age-adjusted) | 4,793.4 | 5,108.9 | 5,412.6 | 5,796.3 | 6,385.5 | 6,872.8 | 7,385.6 | 7,976.9 | 8,493.6 | 9,183.2 |
| **By severity** |  |  |  |  |  |  |  |  |  |  |
| Severe (Grade 1-3) |  |  |  |  |  |  |  |  |  |  |
| Prevalence rate (crude) | 6,186.7 | 6,682.1 | 7,095.4 | 7,557.9 | 7,958.5 | 8,843.9 | 9,428.2 | 10,129.1 | 10,645.7 | 11,271.3 |
| Prevalence rate (age-adjusted) | 8,019.9 | 8,571.8 | 9,073.4 | 9,595.9 | 10,372.0 | 10,961.8 | 11,582.3 | 12,281.6 | 12,870.7 | 13,740.5 |
| Mild (Grade 4-6) |  |  |  |  |  |  |  |  |  |  |
| Prevalence rate (crude) | 1,322.5 | 1,494.2 | 1,667.4 | 1,903.0 | 2,264.6 | 2,604.1 | 2,933.0 | 3,365.1 | 3,682.4 | 4,042.7 |
| Prevalence rate (age-adjusted) | 1,316.6 | 1,443.6 | 1,557.0 | 1,725.4 | 2,012.9 | 2,257.2 | 2,492.7 | 2,820.7 | 3,082.9 | 3,279.7 |
| Grade 1 |  |  |  |  |  |  |  |  |  |  |
| Prevalence rate (crude) | 10,512.0 | 11,223.8 | 11,891.4 | 12,559.8 | 13,594.8 | 14,159.9 | 14,942.0 | 15,686.9 | 16,166.0 | 17,042.3 |
| Prevalence rate (age-adjusted) | 12,937.4 | 13,606.9 | 14,174.5 | 14,783.8 | 15,640.1 | 16,143.3 | 16,770.0 | 17,435.7 | 17,825.9 | 18,886.5 |
| Grade 2 |  |  |  |  |  |  |  |  |  |  |
| Prevalence rate (crude) | 5,729.6 | 6,208.7 | 6,611.0 | 7,113.3 | 7,512.5 | 8,309.1 | 8,768.3 | 9,453.2 | 10,004.6 | 10,546.9 |
| Prevalence rate (age-adjusted) | 7,130.0 | 7,629.8 | 8,117.7 | 8,506.9 | 9,243.7 | 9,773.2 | 10,336.9 | 11,090.1 | 11,814.4 | 12,492.9 |
| Grade 3 |  |  |  |  |  |  |  |  |  |  |
| Prevalence rate (crude) | 3,381.0 | 3,787.6 | 4,060.8 | 4,417.9 | 5,005.0 | 5,621.8 | 6,187.6 | 6,818.9 | 7,326.1 | 7,873.6 |
| Prevalence rate (age-adjusted) | 4,829.3 | 5,301.9 | 5,696.7 | 6,290.1 | 7,000.3 | 7,742.9 | 8,388.9 | 8,996.8 | 9,604.6 | 10,477.7 |
| Grade 4 |  |  |  |  |  |  |  |  |  |  |
| Prevalence rate (crude) | 1,493.0 | 1,669.9 | 1,854.9 | 2,149.9 | 2,567.0 | 2,968.0 | 3,332.7 | 3,887.3 | 4,286.8 | 4,758.7 |
| Prevalence rate (age-adjusted) | 1,886.9 | 2,105.7 | 2,264.2 | 2,422.2 | 2,880.2 | 3,318.3 | 3,398.5 | 3,859.2 | 3,907.4 | 4,234.4 |
| Grade 5 |  |  |  |  |  |  |  |  |  |  |
| Prevalence rate (crude) | 1,391.9 | 1,568.7 | 1,759.1 | 1,995.2 | 2,367.1 | 2,725.3 | 3,061.2 | 3,521.0 | 3,834.6 | 4,172.0 |
| Prevalence rate (age-adjusted) | 1,410.6 | 1,522.2 | 1,625.7 | 1,787.7 | 2,040.8 | 2,196.7 | 2,521.0 | 2,921.7 | 3,283.1 | 3,510.0 |
| Grade 6 |  |  |  |  |  |  |  |  |  |  |
| Prevalence rate (crude) | 1,095.9 | 1,243.3 | 1,386.2 | 1,574.2 | 1,886.0 | 2,164.0 | 2,468.5 | 2,790.0 | 3,055.1 | 3,357.3 |
| Prevalence rate (age-adjusted) | 972.4 | 1,073.3 | 1,179.3 | 1,351.3 | 1,591.4 | 1,819.4 | 2,079.6 | 2,299.5 | 2,585.5 | 2,707.5 |
| **By disability type** |  |  |  |  |  |  |  |  |  |  |
| Physical |  |  |  |  |  |  |  |  |  |  |
| Prevalence rate (crude) | 1,461.1 | 1,650.3 | 1,816.3 | 2,008.9 | 2,318.2 | 2,601.1 | 2,884.4 | 3,259.8 | 3,570.5 | 3,927.3 |
| Prevalence rate (age-adjusted) | 1,794.3 | 1,966.6 | 2,140.8 | 2,326.4 | 2,632.3 | 2,883.8 | 3,129.1 | 3,461.8 | 3,729.6 | 4,109.0 |
| Brain injury |  |  |  |  |  |  |  |  |  |  |
| Prevalence rate (crude) | 5,215.1 | 5,917.7 | 6,402.2 | 6,862.9 | 7,219.7 | 8,089.4 | 8,683.4 | 9,382.8 | 9,817.4 | 10,284.0 |
| Prevalence rate (age-adjusted) | 4,520.2 | 5,025.3 | 5,613.1 | 5,964.1 | 6,558.3 | 6,984.5 | 7,490.7 | 8,074.2 | 8,468.9 | 8,854.4 |
| Communication |  |  |  |  |  |  |  |  |  |  |
| Prevalence rate (crude) | 1,757.5 | 1,968.7 | 2,218.7 | 2,522.8 | 2,952.4 | 3,387.2 | 3,780.0 | 4,276.8 | 4,581.6 | 4,890.6 |
| Prevalence rate (age-adjusted) | 1,807.6 | 1,960.1 | 2,116.9 | 2,282.7 | 2,529.5 | 2,721.9 | 2,939.3 | 3,187.1 | 3,393.3 | 3,592.1 |
| Intellectual |  |  |  |  |  |  |  |  |  |  |
| Prevalence rate (crude) | 21,878.3 | 22,459.1 | 22,870.8 | 23,128.0 | 23.849.8 | 24,312.3 | 24,765.6 | 25,389.5 | 25,803.9 | 26,727.6 |
| Prevalence rate (age-adjusted) | 22,219.5 | 22,722.5 | 23,035.3 | 23,218.1 | 23,930.3 | 24,339.6 | 24,750.4 | 25,377.5 | 25,759.8 | 26,674.4 |
| Major internal organ |  |  |  |  |  |  |  |  |  |  |
| Prevalence rate (crude) | 3,852.4 | 4,286.5 | 4,377.2 | 4,657.1 | 4,952.5 | 5,302.8 | 5,515.4 | 5,820.7 | 5,915.4 | 6,168.0 |
| Prevalence rate (age-adjusted) | 4,014.5 | 4,441.3 | 4,635.8 | 5,024.2 | 5,308.3 | 5,556.1 | 5,604.4 | 5,767.9 | 5,791.5 | 6,126.8 |
